# circINSR Inhibits Adipogenic Differentiation of Adipose-Derived Stromal Vascular Fractions through the miR-152/*MEOX2* Axis in Sheep

**DOI:** 10.3390/ijms24043501

**Published:** 2023-02-09

**Authors:** Bishi Zhao, Hanyue Zhang, Dan Zhao, Yu Liang, Liying Qiao, Jianhua Liu, Yangyang Pan, Kaijie Yang, Wenzhong Liu

**Affiliations:** College of Animal Science, Shanxi Agricultural University, Jinzhong 030801, China

**Keywords:** adipogenesis, circINSR, miR-152, MEOX2, sheep, stromal vascular fraction

## Abstract

Adipose tissue plays a crucial role in energy metabolism. Several studies have shown that circular RNA (circRNA) is involved in the regulation of fat development and lipid metabolism. However, little is known about their involvement in the adipogenic differentiation of ovine stromal vascular fractions (SVFs). Here, based on previous sequencing data and bioinformatics analysis, a novel circINSR was identified in sheep, which acts as a sponge to promote miR-152 in inhibiting the adipogenic differentiation of ovine SVFs. The interactions between circINSR and miR-152 were examined using bioinformatics, luciferase assays, and RNA immunoprecipitation. Of note, we found that circINSR was involved in adipogenic differentiation via the miR-152/mesenchyme homeobox 2 (*MEOX2*) pathway. *MEOX2* inhibited adipogenic differentiation of ovine SVFs and miR-152 inhibited the expression of *MEOX2*. In other words, circINSR directly isolates miR-152 in the cytoplasm and inhibits its ability to promote adipogenic differentiation of ovine SVFs. In summary, this study revealed the role of circINSR in the adipogenic differentiation of ovine SVFs and its regulatory mechanisms, providing a reference for further interpretation of the development of ovine fat and its regulatory mechanisms.

## 1. Introduction

Sheep are domesticated animals with great agricultural value, providing meat and milk to sustain human life [1]. In the process of natural and artificial selection, the degree of fat deposition along the tail vertebra of sheep contributes to the formation of different tail shapes [2]. The tail of fat-tailed sheep, for example, can store energy and help them survive in cold winters, which is an adaptation to the harsh external environment [3]. However, during production, it was found that a large amount of fat deposited in the tail of sheep is not conducive to breeding. Simultaneously, sheep with increased fat deposition consume the mass of the feed, thus reducing the feed conversion rate [4]. Therefore, it is important to explore the mechanism of fat deposition in the tail of sheep to improve production efficiency.

Fat deposition is a complicated process regulated by many genes and non-coding RNAs [5]. Circular RNAs (circRNAs) are a novel class of highly conserved non-coding RNAs characterized by covalently closed loop structures produced by the reverse splicing of genes [6]. Its special structure without 5′ or 3′ polarity is more stable than linear RNA, which is conducive to its biological function [7]. Although circRNAs have a variety of functions [8], they reportedly act as molecular sponges to absorb microRNAs (miRNAs). For instance, circLMO7 acts as a miR-30a-3p sponge to promote gastric cancer progression via the WNT2/β-catenin pathway [9]. CircFGFR4, a competing endogenous RNA (ceRNA), regulates the expression of muscle differentiation-related genes by sponging miR-107 [10]. In addition, circVGLL3 promotes osteogenic differentiation of adipose-derived mesenchymal stem cells (ADSCs) via the miR-326-5p/integrin α5 (Itga5) axis [11]. In adipogenesis, circFLT1 has been reported to facilitate adipocyte differentiation by sponging miR-93 in Qinchuan cattle [12]. In addition, circFUT10 can act as a ceRNA and adsorb let-7 to relieve its inhibitory effects on *PPARGC1B* (peroxisome proliferator-activated receptor γ coactivator 1-β), thus inhibiting the differentiation of bovine adipocytes [13]. However, circRNAs, as miRNA sponges, are rarely reported to be involved in regulating fat deposition in sheep.

With the rapid development of high-throughput sequencing techniques and bioinformatics, many circRNAs have been discovered [14,15]. Preliminary sequencing results from our laboratory screened many unknown circRNAs in sheep. circ0005529, which is located at the second exon of the insulin receptor (*INSR*) gene, was identified and named circINSR. The INSR is an important protein in the insulin signaling pathway and has been reported to be abnormally expressed in the muscles and adipocytes of obese, insulin-resistant, and type 2 diabetic patients [16]. A recent study found an inverse correlation between increasing body mass index (BMI) and decreasing INSR expression in visceral adipose tissue (VAT) in obese humans [17]. The INSR usually functions by binding to insulin [18], and the integrity of INSR signal transduction in adipose tissue is crucial for maintaining lipid metabolism homeostasis [19]. One study found that interference with INSR inhibits the differentiation of porcine adipocytes [20]. Based on these studies, we believe that circINSR may function as an important regulator of adipogenesis in ovine SVFs.

In the present study, the function and molecular mechanism of circINSR during the adipogenic differentiation of ovine SVFs, which are differentially expressed in the tail adipose tissue of Guangling Large-Tailed sheep and Hu sheep, was investigated. The possible regulatory relationships between circINSR, miR-152, and its target gene mesenchyme homeobox 2 (*MEOX2*) were clarified. Mechanistically, circINSR upregulated *MEOX2* by acting as a sponge for miR-152, thereby inhibiting the adipogenic differentiation of ovine SVFs. Collectively, our findings revealed a novel regulatory mechanism of the circINSR/miR-152/*MEOX2* axis in adipogenesis, providing a better understanding of circRNA-regulated adipogenesis. It also contributes to an important reference value for studying the regulation of fat deposition in sheep.

## 2. Results

### 2.1. Identification and Characterization of circINSR in Ovine SVFs

**Figure 1:**
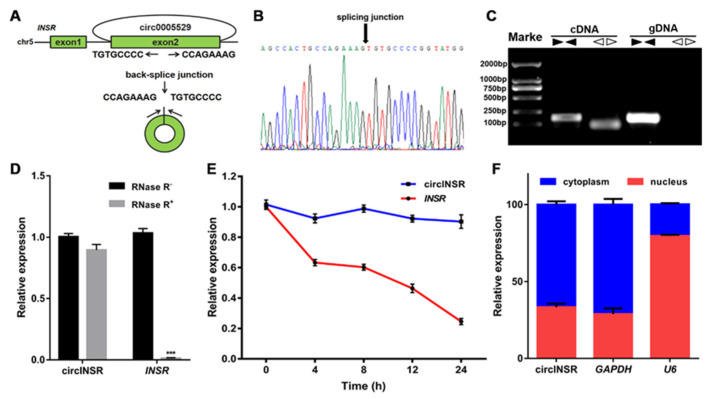
Characterization of circINSR in ovine SVFs. (**A**) circINSR generated from the second exon of the *INSR* gene. (**B**) The existence of circINSR was verified via Sanger sequencing. (**C**) PCR analysis of divergent and convergent primers in cDNA and gDNA. (**D**) The relative expression of circINSR and *INSR* after RNase R treatment. (**E**) The relative expression of circINSR and *INSR* in ovine SVFs treated with ActD at the indicated time point. (**F**) Levels of circINSR in the nuclear and cytoplasmic fractions of ovine SVFs. ***: *p* < 0.001.

A model diagram of the splicing process of circINSR is shown in Figure 1A. To verify the circular structure of circINSR, convergent and divergent primers were designed for circINSR and its corresponding INSR linear transcript. Divergent primers were used to amplify the back-spliced junction of circINSR, which was confirmed by Sanger sequencing (Figure 1B). This sequence was consistent with the RNA sequencing results. Moreover, the results of agarose gel electrophoresis showed that the circular isoform (105 bp fragment) of circINSR was amplified using divergent primers only from cDNA; no amplification product was detected from genomic DNA (gDNA) (Figure 1C). Nevertheless, convergent primers could have produced the linear fragment (180 bp) from both cDNA and gDNA (Figure 1C). circINSR was then characterized by investigating its physical properties. The extracted RNA was treated with RNase R and circINSR was found to be relatively resistant to RNase R digestion compared to linear INSR (Figure 1D). In addition, circINSR was more stable than linear INSR after actinomycin D (ActD—an inhibitor of transcription) treatment (Figure 1E). The intracellular localization of circINSR was identified via real-time quantitative PCR (qRT-PCR) to detect nuclear and cytoplasmic RNA. U6 was used as the nuclear internal reference and glyceraldehyde 3-phosphate (GAPDH) was used as the cytoplasmic internal reference. The results showed that circINSR was preferentially located in the cytoplasm of the ovine SVFs (Figure 1F).

### 2.2. circINSR Inhibits the Adipogenic Differentiation of Ovine SVFs

**Figure 2:**
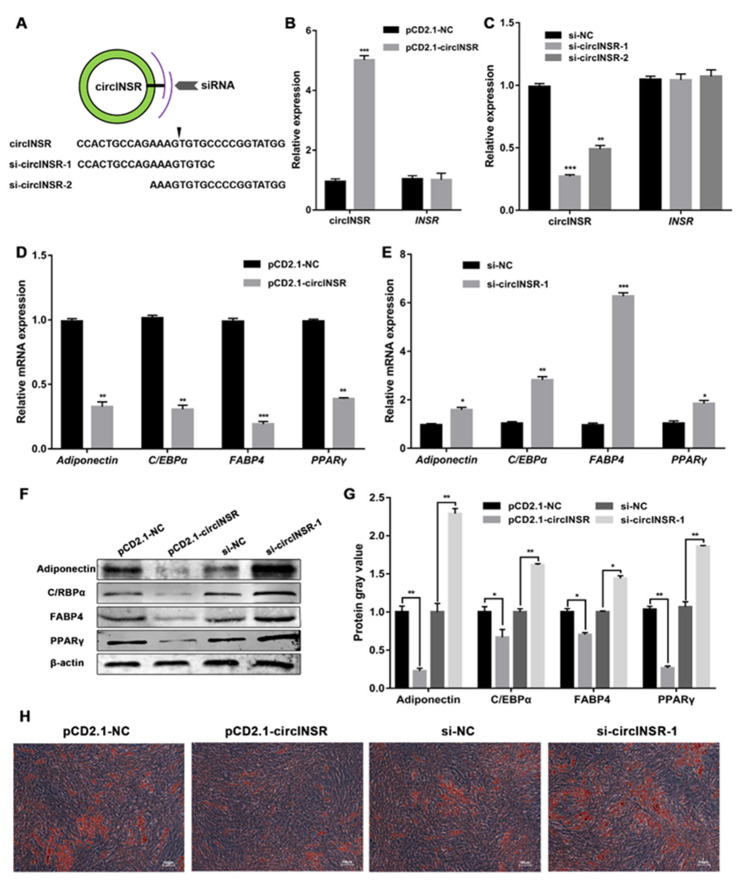
Effects of circINSR on adipogenic differentiation of ovine SVFs. (**A**) Two siRNAs targeting the circINSR junction site were constructed. (**B**) The relative expression of circINSR and INSR mRNA in ovine SVFs after transfection with the circINSR overexpression vector (pCD2.1-circINSR) or the control vector (pCD2.1-NC). (**C**) The relative expression of circINSR and *INSR* mRNA in ovine SVFs after transfection with two siRNAs or the control siRNA (si-NC). (**D**,**E**) The mRNA expression of 4 lipogenic genes in ovine SVFs that overexpress or interfere with circINSR. (**F**,**G**) The protein expression of 4 lipogenic genes in ovine SVFs that overexpress or interfere with circINSR. (**H**) Ovine SVFs were stained with ORO on day 10 after induction of differentiation. Scale bars: 100 μm. *: *p* < 0.05, **: *p* < 0.01, ***: *p* < 0.001.

To explore the physiological function of circINSR during the adipogenic differentiation of ovine SVFs, two siRNAs were designed to target the back-spliced region of circINSR (Figure 2A) and the overexpression vector was constructed. As shown in Figure 2B,C, the overexpression vector and siRNAs of circINSR efficiently increased and reduced the expression of circINSR in ovine SVFs, respectively. Among the two siRNAs, si-circINSR-1 showed a better interference efficiency than si-circINSR-2 and was selected for subsequent experiments. However, RNA expression of linear INSR showed no significant changes. Moreover, qRT-PCR analysis showed that the mRNA expression of the four lipogenic genes was significantly downregulated by circINSR overexpression (Figure 2D) and upregulated by circINSR interference (Figure 2E). We also evaluated the expression of the four lipogenic proteins after overexpression and interference in circINSR (Figure 2F,G) and the expression trends were consistent with those of mRNA. These results suggest that circINSR attenuates adipogenic differentiation of ovine SVFs. In addition, Oil Red O (ORO) staining of the cells showed a significant decrease in lipid droplet formation upon circINSR overexpression (Figure 2H), and circINSR interference enhanced lipid accumulation in adipogenically differentiated ovine SVFs (Figure 2H and Figure S1). Taken together, the results of these experiments indicate that circINSR plays a negative role in the adipogenic differentiation of ovine SVFs.

### 2.3. circINSR Functions as a Sponge for miR-152

**Figure 3:**
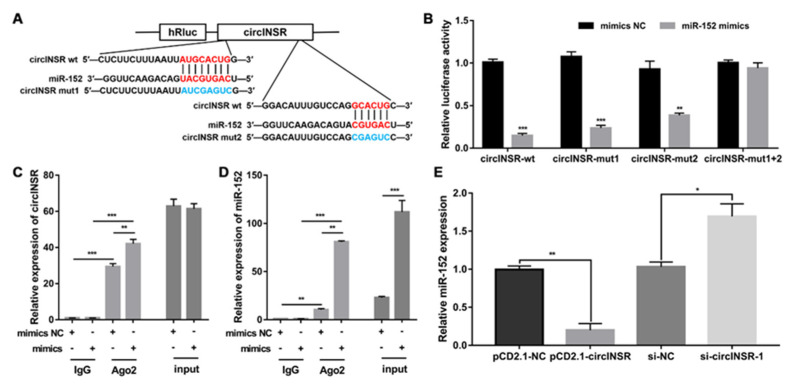
circINSR acts as a sponge for miR-152. (**A**) Schematic illustration of the circINSR-wt, circINSR-mut1, and circINSR-mut2 luciferase vectors. (**B**) The relative luciferase activities were detected in 293 T cells after transfection with circINSR-wt, circINSR-mut1, or circINSR-mut2 and miR152 mimics or mimics NC, respectively. (**C**,**D**) A RIP assay was carried out with anti-Ago2 antibodies or IgG in ovine SVFs after transfection with the miR-152 mimics or mimics NC, and qRT-PCR was then performed to detect the enrichment of circINSR and miR-152. (**E**) The relative expression of miR-152 in ovine SVFs that overexpress or interfere with circINSR. *: *p* < 0.05, **: *p* < 0.01, ***: *p* < 0.001.

Numerous studies have demonstrated that circRNAs act as molecular sponges of miRNAs to regulate the expression of target genes [21,22]. Based on previous sequencing data and bioinformatic predictions, circINSR has two binding sites for miR-152 (Figure 3A). Therefore, we hypothesized that circINSR inhibits adipogenic differentiation of ovine SVFs by sponging miR-152. To validate this hypothesis, luciferase reporter plasmids containing wild-type fragments (circINSR-wt), mutant binding site 1 fragments (circINSR-mut1), mutant binding site 2 fragments (circINSR-mut2), or mutant binding site 1 plus site 2 fragments (circINSR-mut1+2) were used. The miR-152 mimics or NC mimics were co-transfected into 293 T cells with the constructed reporter plasmids. Luciferase activity of circINSR-wt was markedly suppressed (Figure 3B). Meanwhile, the circINSR-mut1 generated lower luciferase activity compared with that generated by circINSR-mut2 (Figure 3B). However, circINSR-mut1+2 co-transfected with miR-152 did not significantly alter the luciferase activity (Figure 3B). These data imply that both binding sites 1 and 2 of circINSR can interact with miR-152 and that binding site 2 has a stronger affinity. To further demonstrate the possibility of this interaction, an RNA immunoprecipitation (RIP) assay using an anti-AGO2 antibody and negative control IgG was performed in ovine SVFs overexpressing miR-152 to limit circINSR and miR-152, and qRT-PCR was performed to analyze the levels of circINSR and miR-152 in the immunoprecipitants. The results showed that both circINSR and miR-152 were more highly expressed in AGO2-containing immunoprecipitants than in IgG immunoprecipitants (Figure 3C,D). Simultaneously, circINSR and miR-152 were dramatically more abundant in ovine SVFs transfected with miR-152 mimics than in ovine SVFs transfected with NC mimics (Figure 3C,D). An increase in miR-152 expression after circINSR downregulation was identified, followed by a decrease after circINSR upregulation (Figure 3E). Collectively, these data suggest that circINSR acts as a ceRNA to sponge miR-152 in ovine SVFs.

### 2.4. miR-152 Promotes the Adipogenic Differentiation of Ovine SVFs

**Figure 4:**
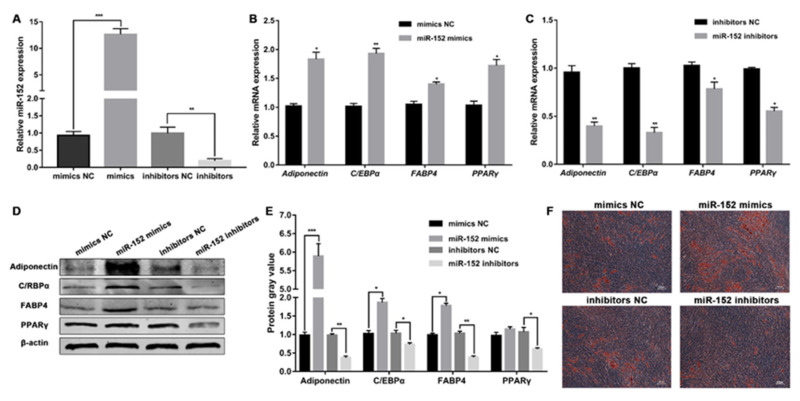
Effects of miR-152 on adipogenic differentiation of ovine SVFs. (**A**) Relative expression of miR-152 after transfection with miR-152 mimics or mimics NC and miR-152 inhibitors or inhibitors NC. (**B**,**C**) The mRNA expression of 4 lipogenic genes in ovine SVFs that overexpress or inhibit miR-152. (**D**,**E**) The protein expression of 4 lipogenic genes in ovine SVFs that overexpress or inhibit miR-152. (**F**) Ovine SVFs were stained with ORO on day 10 after induction of differentiation. Scale bars: 100 μm. *: *p* < 0.05, **: *p* < 0.01, ***: *p* < 0.001.

To investigate whether miR-152 regulates the adipogenic differentiation of ovine SVFs, ovine SVFs at 70% confluence were transfected with miR-152 mimics or miR-152 inhibitors (Figure 4A), and adipogenic differentiation was induced upon reaching full confluence. qRT-PCR and western blot analysis revealed that miR-152 mimics significantly enhanced the expression of Adiponectin, CEBP/α, FABP4, and PPARγ, whereas miR-152 inhibitors obtained the opposite results (Figure 4B–E). Notably, the miR-152 mimics did not significantly increase PPARγ protein expression. We also used ORO staining to observe the effect of miR-152 on adipogenic differentiation of ovine SVFs. The results showed a significant increase in lipid droplet formation upon miR-152 overexpression (Figure 4F). However, fewer lipid droplets were detected in miR-152 inhibitor-transfected ovine SVFs (Figure 4F and Figure S2). These findings revealed that miR-152 facilitates adipogenic differentiation of ovine SVFs, which is accompanied by increased lipid accumulation.

### 2.5. MEOX2 Is Directly Targeted by miR-152 and Indirectly Regulated by circINSR

**Figure 5:**
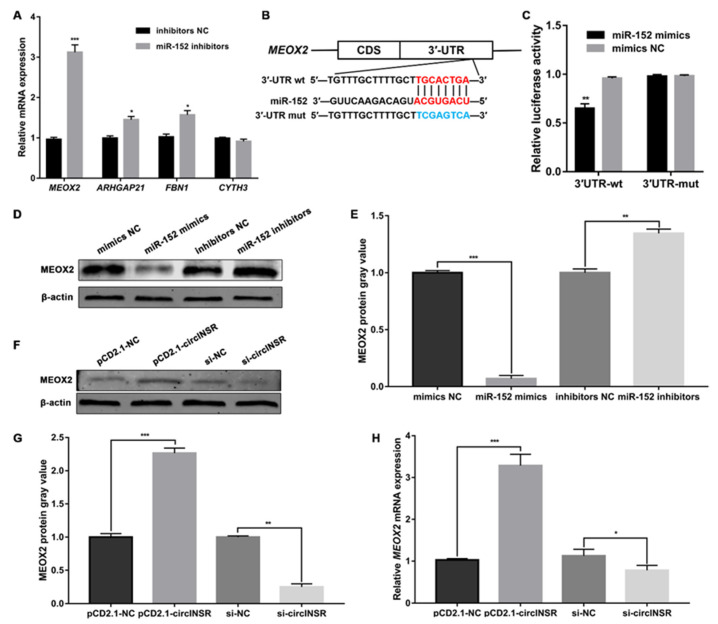
*MEOX2* is directly targeted by miR-152 and indirectly regulated by circINSR. (**A**) The relative mRNA levels of 4 candidate target genes in ovine SVFs that transfect with miR-152 inhibitors or inhibitors NC. (**B**) Schematic illustration of the *MEOX2* 3′-UTR-wt and *MEOX2* 3′-UTR-mut luciferase vectors. (**C**) The relative luciferase activities were detected in 293 T cells after transfection with *MEOX2* 3′-UTR-wt or *MEOX2* 3′-UTR-mut and miR152 mimics or mimics NC, respectively. (**D**,**E**) The protein expression of MEOX2 in ovine SVFs that overexpress or inhibit miR-152. (**F**,**G**) The protein expression of MEOX2 in ovine SVFs that overexpress or interfere with circINSR. (**H**) The mRNA expression of *MEOX2* in ovine SVFs that overexpress or interfere with circINSR. *: *p* < 0.05, **: *p* < 0.01, ***: *p* < 0.001.

To further investigate the downstream genes of miR-152 in ovine SVFs, online bioinformatics was used to identify potential mRNAs that may have binding sites with miR-152. Among the identified potential target genes, we selected four (*MEOX2*, *ARHGAP21*, *FBN1*, *CYTH3*) with high scores that were associated with adipogenesis and had unclear functions. We found that after transfection with miR-152 inhibitors, the expression of *MEOX2* was the most significantly increased compared to the other three genes (Figure 5A). Therefore, we selected *MEOX2* as a target gene of miR-152 for subsequent studies. As shown in Figure 5B, positions 1182–1189 in the *MEOX2* 3′-UTR are potent binding sites for miR-152. To verify this finding, a luciferase reporter plasmid was constructed, which contained either wild-type fragments (*MEOX2* 3′UTR-wt) or mutant binding site fragments (*MEOX2* 3′UTR-mut). Next, the miR-152 mimics or mimics NC were co-transfected into 293 T cells with the constructed reporter plasmids. Compared to mimics NC, co-transfection of miR-152 mimics with *MEOX2* 3′UTR-wt decreased luciferase activity (Figure 5C). However, *MEOX2* 3′UTR-mut co-transfected with miR-152 mimics had no effect on luciferase activity (Figure 5C). Additionally, after transfection with miR-152 mimics, the expression of MEOX2 protein was downregulated (Figure 5D,E). The change in MEOX2 protein expression was consistent with the RNA levels after transfection with miR-152 inhibitors (Figure 5D,E). Next, we detected the expression of *MEOX2* after overexpressing or interference with circINSR in ovine SVFs. qRT-PCR and western blot results showed that *MEOX2* was increased in ovine SVFs with circINSR overexpression, while interference of circINSR decreased *MEOX2* expression (Figure 5F–H). These results suggest that circINSR regulates *MEOX2* by sponging miR-152 in ovine SVFs.

### 2.6. MEOX2 Negatively Regulates the Adipogenic Differentiation of Ovine SVFs

**Figure 6:**
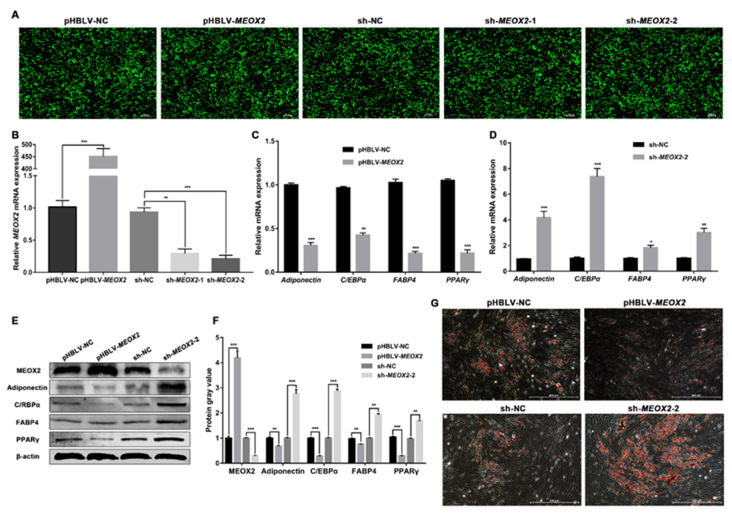
Effects of *MEOX2* on adipogenic differentiation of ovine SVFs. (**A**) Transfection efficiency of overexpression (PHBLV-*MEOX2*) or interference (sh*MEOX2*-1 and sh*MEOX2*-2) with *MEOX2* based on the expression of GFP (green fluorescent protein) in ovine SVFs observed by fluorescence microscopy. Scale bars: 200 μm. (**B**) Relative expression of *MEOX2* after transfection with PHBLV-*MEOX2* or PHBLV-NC and sh*MEOX2*-1, sh*MEOX2*-2, or sh-NC. (**C**,**D**) The mRNA expression of 4 lipogenic genes in ovine SVFs that overexpress or interfere with *MEOX2*. (**E**,**F**) The protein expression of MEOX2 and 4 lipogenic genes in ovine SVFs that overexpress or interfere with *MEOX2*. (**G**) Ovine SVFs were stained with ORO on day 10 after induction of differentiation. Scale bars: 500 μm. *: *p* < 0.05, **: *p* < 0.01, ***: *p* < 0.001.

To confirm the function of *MEOX2* in adipogenically differentiated ovine SVFs, we successfully induced *MEOX2* overexpression and interference using the lentivirus packaging system (Figure 6A). qRT-PCR results also revealed that we successfully overexpressed or interfered with *MEOX2* in ovine SVFs (Figure 6B). Two pairs of oligonucleotide sequences with short hairpin structures (sh*MEOX2*-1 and sh*MEOX2*-2) were designed and synthesized. sh*MEOX2*-2 showed better interference efficiency than sh*MEOX2*-1 and was selected for subsequent experiments. Moreover, we detected the mRNA expression of the four lipogenic genes and found that they were significantly downregulated by *MEOX2* overexpression (Figure 6C) and upregulated by *MEOX2* interference (Figure 6D). We then evaluated the protein expression of MEOX2 and the four lipogenic genes after overexpression and interference with *MEOX2*. The western blot results were consistent with the qRT-PCR results (Figure 6E,F). Correspondingly, ORO staining revealed that the lipid accumulation level of ovine SVFs significantly decreased after overexpressing *MEOX2* (Figure 6G). In contrast, *MEOX2* interference increased lipid accumulation in adipogenically differentiated ovine SVFs (Figure 6G and Figure S3). These data demonstrate that *MEOX2* can suppress adipogenic differentiation of ovine SVFs, which is accompanied by decreased lipid accumulation.

### 2.7. circINSR Represses the Adipogenic Differentiation of Ovine SVFs through the circINSR/mir-152/MEOX2 Axis

**Figure 7:**
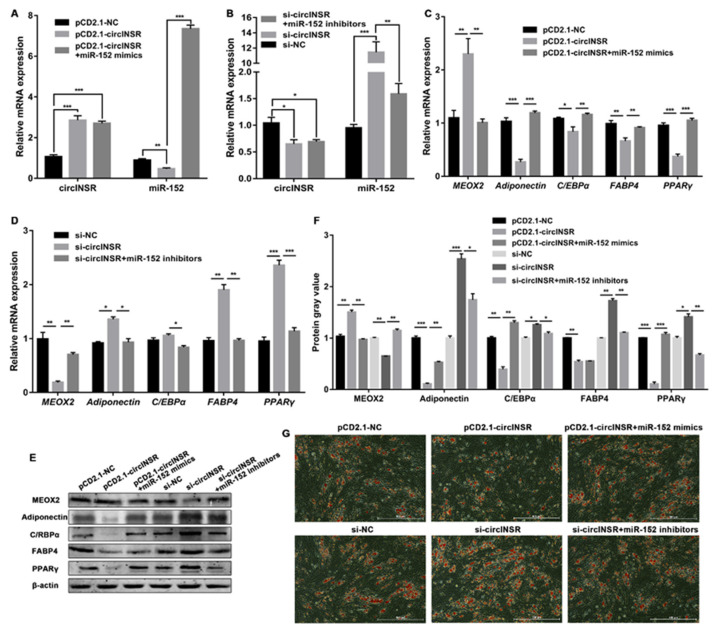
circINSR upregulates the level of *MEOX2* through miR-152. (**A**) The relative expression of circINSR and miR-152 after co-transfection of pCD2.1-circINSR and miR-152 mimics in ovine SVFs. (**B**) The relative expression of circINSR and miR-152 after co-transfection of si-circINSR and miR-152 inhibitors in ovine SVFs. (**C**,**D**) The mRNA expression of *MEOX2* and 4 lipogenic genes in ovine SVFs that co-transfect with pCD2.1-circINSR and miR-152 mimics or si-circINSR and miR-152 inhibitors. (**E**,**F**) The protein expression of MEOX2 and 4 lipogenic genes in ovine SVFs that co-transfect with pCD2.1-circINSR and miR-152 mimics or si-circINSR and miR-152 inhibitors. (**G**) Ovine SVFs were stained with ORO on day 10 after induction of differentiation. Scale bars: 500 μm. *: *p* < 0.05, **: *p* < 0.01, ***: *p* < 0.001.

**Figure 8:**
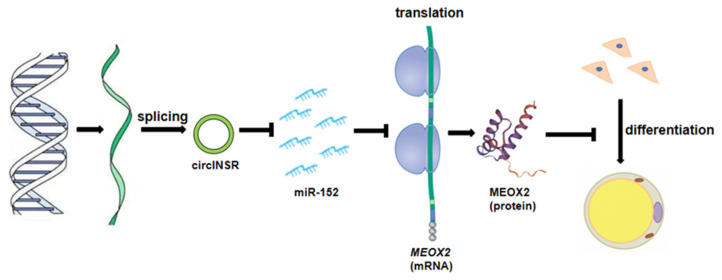
Schematic representation of a model for the major molecular mechanisms of the “circINSR/miR-152/*MEOX2*” axis in ovine SVFs.

To verify whether circINSR represseses the adipogenic differentiation of ovine SVFs through the circINSR/mir-152/*MEOX2* axis, rescue experiments were designed using miR-152 mimics and inhibitors (Figure 7A,B). The qRT-PCR and Western blot analysis showed that upregulation of circINSR enhanced the mRNA and protein level of *MEOX2* and dampened the expression of four lipogenic genes and that the effects caused by overexpressing circINSR could be reversed by miR-152 mimics (miR-152 mimics did not reverse the effect of pCD2.1-circINSR on expression of FABP4 protein) (Figure 7C,E,F). In contrast, downregulation of circINSR decreased the expression of *MEOX2* and increased the expression of the four lipogenic genes both at the mRNA and protein levels, whereas the miR-152 inhibitors overturned these effects (the expression of C/EBPα did not change significantly after the interference of circINSR) (Figure 7D–F). Furthermore, ORO staining showed that overexpression of circINSR significantly reduced the lipid accumulation of ovine SVFs, and this effect was eradicated by miR-152 mimics (Figure 7G). Accordingly, miR-152 inhibitors reversed the adipogenic differentiation effect induced by circINSR interference in ovine SVFs (Figure 7G and Figure S4). In summary, these findings indicate that circINSR may serve as a ceRNA for miR-152 to regulate *MEOX2* expression, negatively regulating the adipogenic differentiation of ovine SVFs (Figure 8).

## 3. Discussion

Adipose development is a complex process affected by many factors [23]. Advancements in bioinformatics techniques and biological technologies have led to the discovery of a growing number of circRNAs participating in this process [24]. It is crucial to elucidate the mechanisms and functions of circRNAs to increase our awareness of the regulatory network involved in ovine adipose development. In this study, we identified a novel circRNA, circINSR, in sheep. Ovine SVFs were used to explore the effect of circINSR on adipogenic differentiation and whether this effect is caused by its role as a miRNA sponge to attenuate the miR-152-mediated inhibition of *MEOX2*.

In this study, we first selected circINSR as the research target from the novel circRNAs with different expressions according to previous sequencing results. Sanger sequencing confirmed that circINSR is derived from the second exon of *INSR* by back-splicing, which is related to adipose tissue development [25]. This is one of the reasons we chose it. Similar to other known circRNAs [26], circINSR is more stable than linear RNA under treatment with RNase R and ActD. A literature review revealed that the function of circRNAs was related to its source gene [8]. The disorder of glucose and lipid metabolism caused by insulin resistance is an important factor leading to obesity [27], and INSR plays an important role in this process. Insulin phosphorylates the insulin receptor substrate through INSR and activates downstream pathways, thereby regulating metabolism [28]. Moreover, the removal of *INSR* in adipocytes results in severe lipid metabolism disorders [19]. Correspondingly, functional gain and loss experiments demonstrated that circINSR inhibited the adipogenic differentiation of ovine SVFs. However, the mechanism by which circINSR functions as a non-coding RNA is unknown.

The function of circRNAs is determined by their location [29]. We found that circINSR was localized predominantly in the cytoplasm, indicating that it may function as a miRNA molecular sponge. Combining bioinformatics analysis and qRT-PCR results, we initially speculated that circINSR is related to miR-152. Dual-luciferase reporter assay and anti-AGO2 RNA immunoprecipitation confirmed that circINSR directly interacts with miR-152. miR-152 belongs to the miR-148/152 family [30] and promotes the differentiation of 3T3-L1 cells [31]. Consistently, our study showed that miR-152 can also facilitate adipogenic differentiation of ovine SVFs. Usually, miRNAs are involved in biological processes through standardized seed region pairing. miR-152 inhibits bovine myoblast proliferation by targeting Kruppel-Like Factor 6 (*KLF6*) [32]. Similarly, miR-148a-3p, a member of the same family, promotes skeletal muscle satellite cell differentiation by targeting *MEOX2* [33]. Bioinformatics analysis indicated that *MEOX2* was also a target gene of miR-152, so we believe that miR-152 may enhance the adipogenic differentiation of ovine SVFs by targeting *MEOX2*. Subsequently, dual-luciferase reporter assay results confirmed that *MEOX2* is a target gene of miR-152 and that miR-152 can downregulate the expression of *MEOX2*, indicating that miR-152 is an important negative regulator of *MEOX2* in ovine SVFs. *MEOX2* was discovered to be a brown adipose-specific transcription factor involved in regulating the differentiation of murine brown preadipocytes [34]. In perivascular adipocytes, *MEOX2* constrained the phosphorylation of ERK1/2 and Akt1/2 proteins, downregulated these signaling molecules and fatty acid synthase (FAS), and decreased lipid content [35]. MEOX2 was also found to impair the effect of chemerin-CMKLR1 on adipogenesis, which promotes adipogenesis through the activation of the Akt/mTOR and ERK pathways [36]. Here, we investigated the function of *MEOX2* during adipogenic differentiation of ovine SVFs using a lentivirus-mediated method to overexpress or interfere with *MEOX2* and found that it can attenuate the adipogenic differentiation of ovine SVFs and accumulation of lipid droplets. Furthermore, we observed that circINSR modulated the expression of MEOX2 at both mRNA and protein levels. These results suggest that circINSR likely regulates *MEOX2* expression by targeting miR-152 and is thus a negative regulator of the adipogenic differentiation of ovine SVFs. However, the pathway through which *MEOX2* regulates adipose differentiation remains unknown. Further research is warranted to address these questions.

To further verify whether circINSR could function via miR-152, we co-transfected pCD2.1-circINSR and miR-152 mimics (or si-circINSR and miR-152 inhibitors) in ovine SVFs. CircINSR overexpression significantly increased MEOX2 expression and repressed the adipogenic differentiation of ovine SVFs, while the functions were reversed following miR-152 overexpression. MEOX2 expression was decreased and lipid accumulation was promoted in ovine SVFs transfected with si-INSR, but these effects were reversed by miR-152 inhibitors. The results of the co-transfection experiments showed that circINSR acted as a decoy to sequester miR-152 to alleviate the suppression of its target gene *MEOX2* to inhibit the adipogenic differentiation of ovine SVFs. However, we are aware of a shortcoming of this study: miR-152 and MEOX2 are only two of the possible targets through which circINSR functions. CircINSR may also be related to other miRNAs or pathways, which warrants further study.

In addition, we noted inconsistent mRNA and protein expression of some genes in this study, such as PPARγ in miR-152 functional verification, and FABP4 and *C/EBPα* in the rescue experiment. By reviewing the literature, this result was found to be understandable. There is a space and time gap between transcription and translation of eukaryotic genes [37,38]. It is possible that protein expression is still increasing when the mRNA peaks or that the mRNA is degraded by the time the protein peaks. Post-transcription processing involves the degradation of transcription products and post-translational modifications [39,40]. Therefore, the transcription and translation levels are not exactly the same.

In conclusion, circINSR acts as a sponge for miR-152 and triggers the promotion of *MEOX2* expression and inhibition of adipogenic differentiation in ovine SVFs. Our study improves the regulatory network of ovine fat development, providing a basis for further research on the metabolic mechanisms of ovine fat.

## 4. Materials and Methods

### 4.1. Ethics Statement

All animal procedures were approved by the Animal Care and Ethical Committee of Shanxi Agricultural University, China.

### 4.2. Isolation and Culture of Ovine SVFs

Ovine SVFs were isolated from the tail adipose tissues of 3-day-old lambs. The tissue was rinsed, minced, and digested with 2 mg/mL collagenase type II (Solarbio, Beijing, China) at 37 °C for 45 min. The digested cell fractions were subsequently centrifuged at 1000× *g* for 10 min. The pellets were resuspended in a growth medium containing 89% low-glucose Dulbecco’s modified Eagle’s medium (DMEM, Biological Industries, Kibbutz Beit Haemek, Israel), 10% fetal bovine serum (FBS, Biological Industries, Kibbutz Beit Haemek, Israel), and 1% penicillin-streptomycin (10,000 U/mL of penicillin and 10,000 μg/mL streptomycin; Gibco, Waltham, MA, USA). After filtration through 75 and 37.5 μm nylon meshes, the filtrate was inoculated into a 60 mm petri dish. The obtained cells were cultured in a growth medium in a 5% CO_2_ incubator at 37 °C. The culture medium was changed every 24 h.

### 4.3. Adipogenic Differentiation and Oil Red O Staining

After the cells reached 100% confluence, they were induced using a differentiation medium consisting of 10 mM rosiglitazone (Cayman Chemical, Ann Arbor, MI, USA), low-glucose DMEM, 10% FBS, 1.4 mg/mL 3-isobutyl-1-methylxanthine (Solarbio, Beijing, China), 1 mg/mL dexamethasone (Solarbio, Beijing, China), and 3 mg/mL bovine insulin (Solarbio, Beijing, China). The medium was changed every two days. After 4 days of induction, the medium was replaced with a maintenance medium containing low-glucose DMEM, 10% FBS, 3 mg/mL bovine insulin, and 10 mM rosiglitazone. The medium was replaced every 2 days.

To stain lipids, the cells were washed with ice-cold PBS (Solarbio, Beijing, China) three times and fixed overnight in 4% paraformaldehyde at 4 °C. The cells were then incubated with ORO working solution (Solarbio, Beijing, China) for 30 min. After washing 3 times with double-distilled water, images were captured using a microscope (Leica, Wetzlar, Germany). The percentage of differentiated SVF cells containing lipid droplets was analyzed using ImageJ software, version 1.48 (U.S. National Institutes of Health, Bethesda, MD, USA).

### 4.4. RNA, Genomic DNA (gDNA) Extraction, and Real-Time Quantitative PCR (qRT-PCR)

Total RNA was extracted from cells using RNAiso Plus (Takara, Kusatsu, Japan). gDNA was extracted using DNAiso Reagent (Takara, Kusatsu, Japan) according to the manufacturer’s instructions. Nuclear and cytoplasmic fractions were extracted using the PARIS kit (Thermo Fisher Scientific, Waltham, MA, USA). The RNA concentration and integrity were determined by spectrophotometry using a Nanodrop 2000 spectrophotometer (Thermo Fisher Scientific, Waltham, MA, USA) and 1% agarose gel electrophoresis, respectively. RNA was reverse-transcribed using a PrimeScript RT reagent kit with gDNA Eraser (Perfect Real Time, Takara, Kusatsu, Japan) or miRcute Plus miRNA First-Strand cDNA kit (Tiangen, Beijing, China). qRT-PCR was performed on a Bio-Rad CFX Connect Real-Time System (Bio-Rad, Hercules, CA, USA) using the TB Green Premix Ex Taq II master mix (Takara, Kusatsu, Japan) or the miRcute Plus miRNA qPCR kit (Tiangen, Beijing, China). Based on the circINSR sequence, divergent and convergent primers were designed to determine its authenticity. The expression of all coding genes was normalized to that of β-actin and U6 small RNA was used as an internal reference to evaluate the level of miR-152. The 2^−ΔΔCt^ method was used to estimate the relative expression levels. All primer sequences used in this experiment are listed in Table 1 and Table 2.

### 4.5. Vector Construction and Cell Transfection

Full-length circINSR was cloned to construct the pCD2.1-circINSR overexpression vector (Geneseed, Guangzhou, China), while the mock vector with no circINSR sequence served as a control. siRNAs targeting the back-splice junction site of circINSR and si-NC were synthesized by RiboBio (Geneseed, Guangzhou, China) and their efficiency was detected by qRT-PCR. The mimics and inhibitors of miR-152 were purchased from Sangon (Shanghai, China). Lipofectamine 3000 (Thermo Fisher Scientific, Waltham, MA, USA) was used for cell transfection. The siRNA sequences of circINSR are listed in Table 3.

### 4.6. Lentivirus Infection

The encoding sequence of *MEOX2* was ligated into the pHBLV-CMIVE ZsGreen-T2A-puro vector (Hanbio, Shanghai, China). We designed and synthesized two pairs of oligonucleotide sequences with short hairpin structures (sh*MEOX2*-1 and sh*MEOX2*-2), targeting the coding region of the *MEOX2* gene. These oligonucleotides were then heat-treated to produce complementary double strands that were attached to the pHBLV-U6-ZsGreen-T2A-puro plasmid (Hanbio, Shanghai, China) between the BamHI and EcoRI restriction sites. The shRNA sequences for *MEOX2* are shown in Table 4. The recombinant plasmid and two packaged plasmids, PMD2.g and psPAX2 (both purchased from Hanbio, Shanghai, China), were transferred to 293 T cells. Forty-eight hours after transfection, the supernatant was collected and filtered through a 0.45-μm filter membrane. The filtrate was collected in a centrifuge tube. When the ovine SVFs density reached approximately 60%, recombinant lentivirus was used to transfect ovine SVFs. The medium was changed 24 h after infection. The cells in each group were collected 12 days after differentiation and subjected to qRT-PCR, western blotting, and ORO staining.

### 4.7. Western Blot

Proteins were isolated from ovine SVFs using a lysis buffer supplemented with protease inhibitors (Solarbio, Beijing, China), phosphatase inhibitors (Solarbio, Beijing, China), and phenylmethylsulfonyl fluoride (PMSF, Solarbio, Beijing, China). Equal amounts of protein were separated by SDS-PAGE, transferred to nitrocellulose filter membranes (Solarbio, Beijing, China), and sealed in 5% skim milk for 1 h. Membranes were then incubated with primary antibodies (anti-β-actin: Immunoway, Beijing, China; anti-MEOX2: Proteintech, Wuhan, China; anti-adiponectin: Proteintech, Wuhan, China; anti-C/EBPα: Proteintech, Wuhan, China; anti-FABP4: Proteintech, Wuhan, China; anti-PPARγ: Proteintech, Wuhan, China) and secondary antibodies (LI-COR, Lincoln, NE, USA). After washing three times, the membranes were imaged using an Odyssey Clx imaging system (LI-COR, Lincoln, NE, USA).

### 4.8. RNase R Treatment

Total RNA was incubated with or without 5 U/µg RNase R (Geneseed, Guangzhou, China) for 15 min at 37 °C. Next, the abundance of linear and circular RNA was analyzed using qRT-PCR.

### 4.9. Actinomycin D Assay

Ovine SVFs were exposed to 2 µg/mL ActD (MilliporeSigma, Burlington, MA, USA) for 0, 4, 8, 12, and 24 h. Total RNA was then extracted to test the stability of linear RNA and circular RNA using qRT-PCR.

### 4.10. Dual-Luciferase Reporter Assay

The wild-type (WT) sequence of circINSR, the *MEOX2* 3′ untranslated region (3′-UTR), and the indicated mutant circINSR and *MEOX2* 3′-UTR (Mut) containing the predicted miR-152 binding site were synthesized. It was cloned into the double-luciferase reporter vector pmirGLO (Promega, Shanghai, China). The resulting double-luciferase reporter plasmid (WT or Mut) was co-transfected into 293 T cells with miR-152 mimics or miR-152 mimics NC using Lipofectamine 3000. After 48 h of incubation, the relative relationship between firefly luciferase activity and the corresponding Renilla luciferase activity was measured and analyzed using a dual luciferase assay system (Promega, Shanghai, China) according to the manufacturer’s protocol.

### 4.11. RIP

The RIP assay was conducted using an RNA Immunoprecipitation Kit (BersinBio, Guangzhou, China) according to the manufacturer’s instructions. Ovine SVFs were transfected with miR-152 mimics or mimics NC, and 1 × 10^7^ cells were collected for the RIP assay. In this experiment, an anti-Ago2 antibody (BOSTER, Wuhan, China) or a negative control IgG was used. The RNA in the immunoprecipitated product was extracted, and the co-precipitated RNA was used to evaluate the abundance of circINSR and miR-152 by qRT-PCR.

### 4.12. Statistical Analysis

Statistical analysis was performed using GraphPad Prism software (version 7.0; GraphPad, San Diego, CA, USA) and is presented as the mean ± SEM. A Student’s *t* test was used to assess differences between the experimental group and control group, and a one-way ANOVA was used for comparisons among multiple groups. For all analyses, *p* < 0.05 was considered statistically significant.

## Figures and Tables

**Table 1:** The qPCR primers for application of mRNAs.

Name of Primers	GenBank Accession Number	Sequences (5′-3′)
circINSR forward		CTGCCACTGTCATCAACGG
circINSR reverse		GCAGCCGGGTGAGATTATTC
*PPARγ* forward	NM_001100921.1	ATCTTGACGGGAAAGACGAC
*PPARγ* reverse		AAACTGACACCCCTGGAAGAT
*FABP*4 forward	NM_001114667.1	AAACTGGGATGGGAAATCAACC
*FABP*4 reverse		TGCTCTCTCGTAAACTCTGGTAGC
*Adiponectin* forward	NM_001308565.1	ATCCCCGGGCTGTACTACTT
*Adiponectin* reverse		CTGGTCCACGTTCTGGTTCT
*C/EBPα* forward	NM_001308574.1	TCCGTGGACAAGAACAGCAA
*C/EBPα* reverse		TCATTGTCACTGGTCAGCTCC
*β-Actin* forward	NM_001009784.3	TGATGATATTGCTGCGCTCG
*β-Actin* reverse		GGGTCAGGATGCCTCTCTTG
miR-152		GCGCGCTCAGTGCATGACAGAAC
*U6* forward	XR_003587591.1	CTCGCTTCGGCAGCACA
*U6* reverse		AACGCTTCACGAATTTGCGT
*MEOX2* forward	XM_004007766.5	AGCGATAGCTCAGACTCCCA
*MEOX2* reverse		TCGCCTCAGTCTGGTCAGATA
*ARHGAP21* forward	ARHGAP21	GCGCTTGCTCCGGAAAGAT
*ARHGAP21* reverse		CTCCTGTGCAGCTAGTGAGG
*FBN1* forward	XM_012181624.4	GGCAAACGGTTTTTCAAAGACAT
*FBN1* reverse		TCCAGAGCAAAGCCGCTATC
*CYTH3* forward	XM_042240307.1	GGATGAAGTCCATCAGAGCGA
*CYTH3* reverse		ACTCTGCTGTGGGGTTTCAG

**Table 2:** Sequences of the divergent and convergent primers for circINSR.

Name	Sequences (5′-3′)
circINSR convergent	F: TGGAGAGCCTGAAGGACTTG
	R: CCAGGTAGCAGAGTTCATTGTT
circINSR divergent	F: CTGCCACTGTCATCAACGG
	R: GCAGCCGGGTGAGATTATTC

**Table 3:** siRNA sequences of circINSR.

Name	Sequences (5′-3′)
si-circINSR-1	CCACTGCCAGAAAGTGTGC
si-circINSR-2	AAAGTGTGCCCCGGTATGG

**Table 4:** shRNA sequences for *MEOX2*.

Names	Sequences (5′-3′)
sh-*MEOX2*-1 forward	GATCCGCAGAATTTGCCCATCATAATTTCAAGAGAATTATGATGGGCAAATTCTGCTTTTTTG
sh-*MEOX2*-1 reverse	AATTCAAAAAAGCAGAATTTGCCCATCATAATTCTCTTGAAATTATGATGGGCAAATTCTGCG
sh-*MEOX2*-2 forward	GATCCGCTCTGAGCATGCACACTTATTTCAAGAGAATAAGTGTGCATGCTCAGAGCTTTTTTG
sh-*MEOX2*-2 reverse	AATTCAAAAAAGCTCTGAGCATGCACACTTATTCTCTTGAAATAAGTGTGCATGCTCAGAGCG

## Data Availability

All data generated or analyzed during this study are included in this published article. The data that support the findings of this study are available from the corresponding author upon request.

## Supplementary Materials

The supporting information can be downloaded at: https://www.mdpi.com/article/10.3390/ijms24043501/s1.
