# Supplementary material for: circINSR Inhibits Adipogenic Differentiation of Adipose-Derived Stromal Vascular Fractions through the miR-152/MEOX2 Axis in Sheep
Source: Int J Mol Sci. 2023 Feb 9;24(4):3501. doi: 10.3390/ijms24043501 (PMC9964708; doi:10.3390/ijms24043501)
Supplement: Supplementary file 1 [file ijms-24-03501-s001.zip › ijms-2203405-supplementary.pdf]

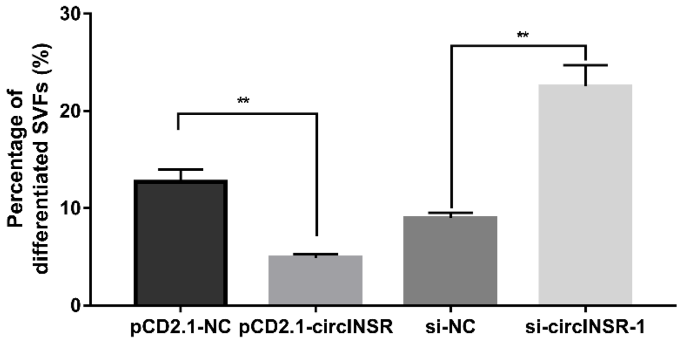

Figure S1. The percentage of differentiated SVFs containing lipid droplets that overexpress or interfere with circINSR.

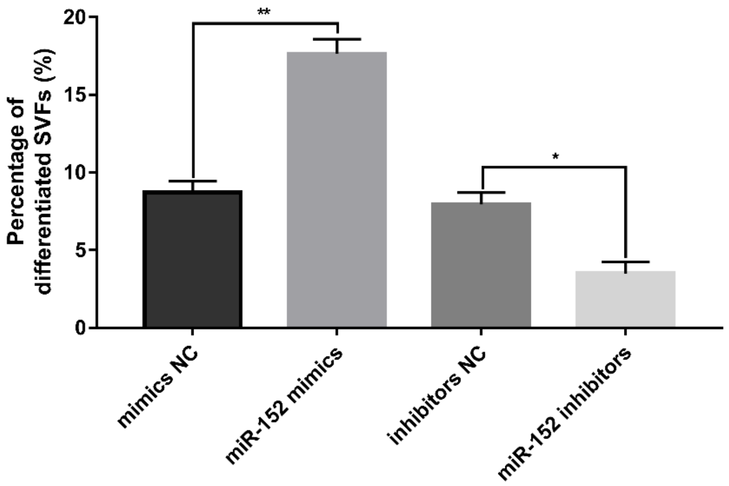

Figure S2. The percentage of differentiated SVFs containing lipid droplets that overexpress or inhibit miR-152.

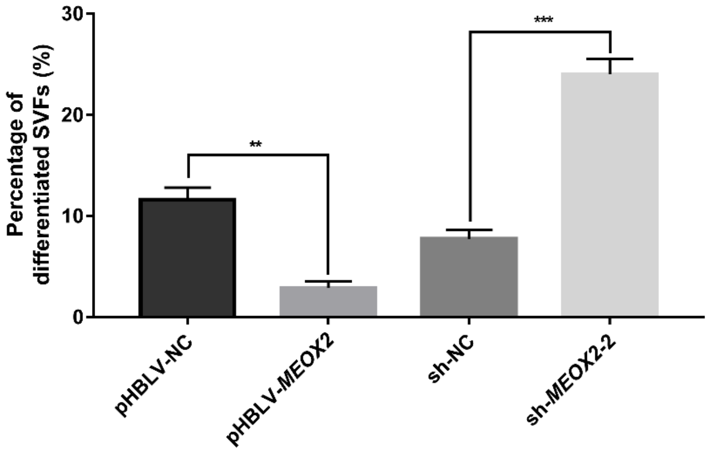

Figure S3. The percentage of differentiated SVFs containing lipid droplets that overexpress or interfere with *MEOX2*.

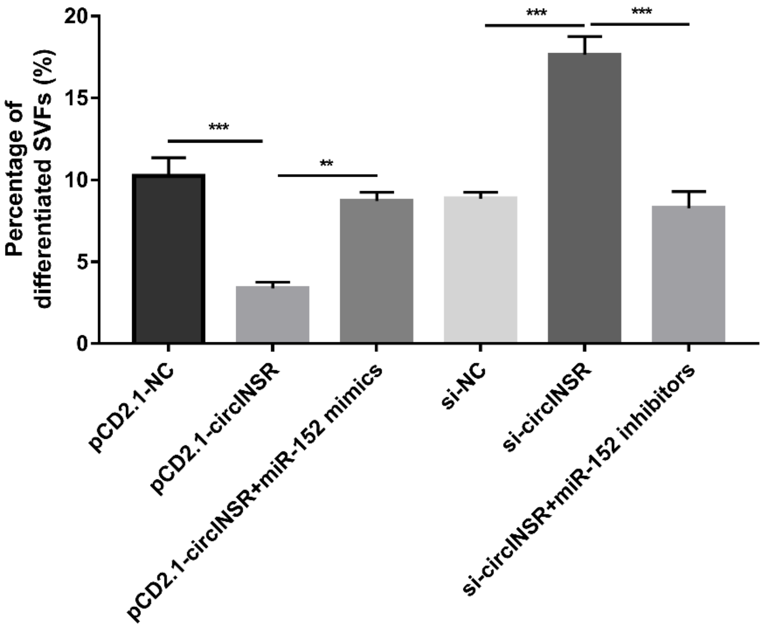

Figure S4. The percentage of differentiated SVFs containing lipid droplets that co-transfect with pCD2.1-circINSR and miR-152 mimics or si-circINSR and miR-152 inhibitors.
